# Turmeric Polymer Fiber Mats (T-PFMs): Solution Blow Spun Turmeric/PLGA Composites as a Comprehensive Wound Care Platform

**DOI:** 10.1021/acsapm.5c04766

**Published:** 2026-03-24

**Authors:** Kishan Kalluraya Yogesh, Shannon Killen, Andrew Mancuso, Christina Viso, Joy Nasr, Kehinde Aiyegboyin, Victoria Cannava, Zaghloul Ahmed, Krishnaswami S Raja

**Affiliations:** † Department of Chemistry, The College of Staten Island, 14771 City University of New York, 2800 Victory Blvd, Staten Island, New York 10314, United States; ‡ Graduate Center, City University of New York, 365 fifth Ave, New York, New York 10016, United States; § Department of Physical Therapy, The College of Staten Island, City University of New York, 2800 Victory Blvd, Staten Island, New York 10314, United States; ∥ Center for Developmental Neuroscience, The College of Staten Island, City University of New York, 2800 Victory Blvd, Staten Island, New York 10314, United States; ⊥ Institute for Macromolecular Assemblies, The College of Staten Island, City University of New York, 2800 Victory Blvd, Staten Island, New York 10314, United States; # Advanced Science Research Center Nanoscience Program, City University of New York, 85 St Nicholas Terrace, New York, New York 10031, United States

**Keywords:** Turmeric, Polymer Fiber Mats, Wound Healing, Hemostatic, PLGA, Bioresorbable, Solution Blow Spinning

## Abstract

This work lays the groundwork to transform turmeric, a historically used natural wound remedy, into a modern, comprehensive wound care and hemostatic biomaterial in the form of solution blow-spun, bioresorbable turmeric/PLGA polymer fiber mats (PFMs). Ultrafine polymer mats were produced using a bottom-loading, siphon-fed airbrush to spray a turmeric/PLGA solution in acetone, enabling rapid and uniform fiber deposition onto a surface. Optimal turmeric loading in the PFM was identified based on dynamic mechanical analysis and surface wettability, with 0.5% (w/v) tur/PLGA selected for further evaluation. The optimized PFMs were then characterized to evaluate water uptake capacity, degradation kinetics, and surface morphology. Turmeric-loaded PFMs inhibited the growth of *Escherichia coli* and *Staphylococcus aureus* and promoted ∼30% faster wound contraction, greater re-epithelialization, and well-organized, mature collagen deposition compared to controls in a murine skin excisional wound model. A pilot mouse tail-vein amputation bleeding model further demonstrated the potential of turmeric-PFMs to be used as a hemostatic dressing. This report lays the foundation for the development of a bioresorbable, turmeric-based, comprehensive wound care platform.

## Graphical abstract

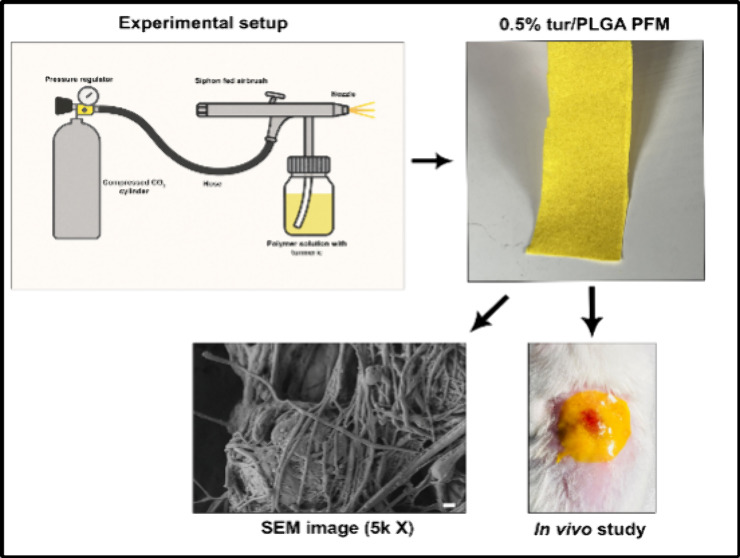

## 1 Introduction

From the dawn of civilization, humanity has used naturally occurring materials such as turmeric and medicinal clay to treat wounds and other epidermal conditions.^1^ While several clay-based wound care products have progressed into modern clinical use, there are currently no commercially available or actively developing wound care technologies that utilize turmeric powder as the primary bioactive agent. To the best of our knowledge, the PLGA–turmeric composite bioresorbable polymer fiber mats (PFMs) described in this work represent the first contemporary wound care platform leveraging turmeric as the active component which can also be used as a potential hemostatic gauze. Turmeric powder, derived from the dried rhizomes of *Curcuma longa*, has been used for millennia across Asia as a spice, cosmetic, dye, and utilized as a therapeutic ingredient in traditional medical systems such as Ayurveda.^2^ Archaeological evidence of turmeric has been found at sites of the Sindhu-Saraswati Civilization (Indus Valley) in Northwest India dating to more than 2000 BCE.^3^

Ancient medicines need to be modernized through creative formulation chemistry to transform them into practical products. A classic example is QuikClot Combat Gauze (QCCG), a clay-based hemostatic dressing widely used to stop bleeding and credited with saving countless lives. However, QCCG is not bioresorbable, provides no enhancement to wound healing, and its residual particles have been implicated in adverse outcomes such as coagulopathy, progressive shock, and embolus formation.^4^

This work follows the principle that product leads developed using components already FDA-approved or with long use history in humans can reduce development risks due to prior known toxicity profiles thereby increasing the probability of success at the clinical stage. Wound healing and hemostasis are complex biological processes; it is not surprising that ancient treatments relied on complex heterogeneous mixtures.^5^ Turmeric powder is a chemically diverse mixture composed of 60–70% carbohydrates, 6–13% water, 6–8% protein, 5–10% fat, 3–7% mineral matter, 3–7% essential oils (including turmerone and germacrone), 2–7% fiber, and 1–6% curcuminoids such as curcumin, dimethoxycurcumin, and bisdimethoxycurcumin.^6^

Formulating turmeric is challenging because its components span a broad range of solubilities: some dissolve in organic solvents and fats, others are insoluble, and several are sparingly soluble in water.^7,8^ As a result, academic studies often take a reductionist approach and use only curcumin, despite the fact that isolated curcumin is anticoagulant and promotes bleeding.^9^ In contrast, traditional systems such as Ayurveda and Traditional Chinese Medicine have always relied on whole turmeric powder rather than isolated curcumin.^10,11^ Therefore, this historical use of turmeric powder for wound care provides an ethnopharmacological basis for investigating its efficacy in wound treatment. Turmeric powder is also dramatically less expensive than purified curcumin, making it more cost-effective.

Curcumin itself has generated intense research interest due to anti-inflammatory,^12^ antioxidant,^13^ antibacterial^14^ and wound healing properties.^15,16^ It has been evaluated against cancers,^17,18^ Alzheimer’s disease,^19^ cystic fibrosis,^20^ Crohn’s disease^21^ and rheumatoid arthritis.^22^ Multiple studies show that curcumin application enhances epithelial regeneration, fibroblast proliferation, and vascularization.^15,23^ However, curcumin has extremely low water solubility, low stability, and limited bioavailability.^24^ Contributing factors include inadequate absorption, rapid metabolism, degradation, and fast clearance.^24,25^ Consequently, only small amounts reach plasma or target tissues. There is a considerable body of literature including our previous work which has been focused on improving the bioavailability of curcumin.^19,26^ Some of these strategies include, preparation of nanoemulsions,^27^ silica based carriers,^28^ other nanoparticles,^15^ chemical derivatives,^26^ bioconjugates,^29^ hydrogels^30^ and polymer composites.^31^

This work addresses a longstanding formulation challenge by developing a wound care platform that employs whole turmeric powder and can potentially also serve as a hemostatic gauze. To maximize the therapeutic potential of turmeric and improve curcumin bioavailability, a solution blow spinning (SBS) method was used to fabricate biodegradable PLGA fiber mats containing turmeric powder. These bioactive polymeric fiber mats (PFMs) can be applied directly to wounds to seal the site, promote healing, and reduce bacterial burden. SBS is a novel fiber fabrication technique that has shown considerable potential for applications in surgery, drug delivery, and tissue engineering.^32–34^ In one reported study, airbrushed nanofibers effectively stopped bleeding and air leakage following a liver injury.^35^

The PFMs presented herein could be deposited onto a glass substrate and peeled off as standalone bioactive bandages or sprayed directly onto a bleeding surface. Here, we focus on the bandage format. Turmeric-loaded PFMs with varying concentrations were characterized for their mechanical properties and surface wettability using dynamic mechanical analysis and contact angle measurements. The optimal formulation was then evaluated for water retention, degradation behavior, surface morphology, *in vivo* wound healing efficacy in a murine model, antibacterial activity, and histology. The results support the potential of turmeric-loaded PFMs as a comprehensive, bioresorbable wound care platform. A pilot murine tail vein amputation bleeding study assessed the hemostatic potential of the PFMs.

## 2 Methods

### 2.1 Preparation of Polymer Fiber Mats by Solution Blow Spinning

Polymer fiber mats (PFMs) were fabricated using an airbrush system (Grex Genesis.XBi, 0.5 mm nozzle diameter) connected to a compressed CO_2_ tank equipped with a precision pressure regulator. The operating pressure was maintained at 70 PSI. Polymer solutions were sprayed onto 75 mm × 25 mm glass microscope slides, with the nozzle positioned approximately 25 cm from the substrate. Rapid evaporation of the volatile solvent resulted in the deposition of ultrafine polymer fibers, forming nonwoven PFMs.

All polymer solutions consisted of 10% (w/v) PLGA (inherent viscosity 0.81 dL/g in hexafluoroisopropanol, Mw 63 kDa, 50:50 lactide:glycolide; Lactel, Evonik Industries) dissolved in acetone. Commercially available turmeric powder was added to the polymer solution at concentrations of 0.1%, 0.5%, and 2.5% (w/v) and magnetically stirred at room temperature for 10 min to obtain polymer solutions with varying turmeric content and immediately blow spun. The resulting PFMs were peeled from the glass substrates and stored under vacuum until further use. Prior to the *in vivo* experiments, the mats were disinfected by UV irradiation.

### 2.2 Dynamic Mechanical Analysis of the PFMs

Mechanical properties of the PFMs containing different concentrations of turmeric powder (0.1%, 0.5%, and 2.5%) as well as plain PLGA PFMs were evaluated by dynamic mechanical analysis. PFM samples were cut into 20 × 10 mm specimens, and tested on a Dynamic Mechanical Analyzer (Q800, TA Instruments) with a film tension clamp. Tensile tests were performed under a 0.001 N preload at 1%/min strain rate until failure (n = 4). PFM thickness was measured with a screw gauge prior to testing.

### 2.3 Surface Wettability of the PFMs

Surface wettability of the PFMs containing different concentrations of turmeric powder (0.1%, 0.5%, and 2.5%) as well as plain PLGA PFMs were evaluated by contact angle measurements. Measurements were performed using a standard goniometer (Model 250-F1, rame-hart Instrument Co., USA). A 15 μL droplet of phosphate buffer solution (PBS) was dispensed onto the PFM surface, and both the initial contact angle and the contact angle after 60 s were recorded. The same procedure was repeated for a solution of hemoglobin (1 mg/mL) supplemented with bovine serum albumin (1 mg/mL) in PBS (HGB solution).

### 2.4 Water Retention Capacity of the PFMs

The water retention of the plain PLGA PFMs and 0.5% turmeric/PLGA PFMs was evaluated by gravimetric method in accordance with the standard literature protocol. Preweighed dry PFM samples were placed in microcentrifuge tubes and subsequently submerged in PBS. At predetermined time intervals (1, 3, 6, 24, and 48 h.) the samples were removed, gently blotted with filter paper to remove excess surface liquid and immediately weighed (*n* = 6). The water content (%) was calculated using the following equation.

$$\mathrm{Water Content}\;\left(\%\right)=\left(\left(W_{T}-W_{0}\right)/W_{0}\right)\times 100$$

where *W*_0_ = weight of the dry sample and *W*_T_ = weight of the sample after immersion at time *T*.

### 2.5 Degradation Behavior

The degradation of the plain PLGA PFMs and the 0.5% turmeric/PLGA PFMs were analyzed by gel permeation chromatography (GPC). Samples (15 mg) were submerged in 15 mL PBS and shaken at 37 °C in a shaker incubator operating at 60 rpm for 45 days. The PBS was refreshed every 24 h. At predetermined time points (0, 1, 7, 14, 21, 30, and 45 days), samples (n = 3) were collected, removed from PBS, and dried overnight in a vacuum oven. Number-average (Mn) and weight-average (Mw) molecular weights of the samples were determined by GPC using a Waters 515 HPLC pump with an in-line degasser, a Waters 2410 refractive index detector, and Agilent columns in series. Samples were dissolved at 1 mg/mL in tetrahydrofuran (THF), which also served as the eluent at a flow rate of 1 mL/min. Molecular weights were reported as poly­(styrene)-equivalent values using a calibration curve generated from EasiCal polystyrene standards (Agilent).

### 2.6 Surface Morphology and Fiber Diameter

The morphological characteristics of the PFMs were examined using a scanning electron microscope (Zeiss Merlin VP Compact, Zeiss) operated at 5 kV in secondary electron mode. Prior to imaging, the samples were sputter-coated with a thin layer of gold–palladium to enhance conductivity. Fiber diameters were quantified using ImageJ software (NIH, USA) based on 60 randomly selected measurements per sample.

### 2.7 Antibacterial Evaluation of the PFMs

The antibacterial activity of the 0.5% tur/PLGA PFMs and plain PLGA PFMs were evaluated against *Escherichia coli* (ATCC 25922) and *Staphylococcus aureus* (ATCC 25923). Bacterial cultures were grown overnight in Lauria broth, and the overnight culture was used to prepare the log phase bacteria. The log phase bacterial solution was approximately diluted to 1 × 10^5^ CFU/mL via optical density measurement. Sterilized PFM samples were cut into small pieces and added to the bacterial suspensions in broth at a final concentration of 25 mg/mL. Plain PLGA PFMs and cultures without PFMs were used as controls. The suspensions were incubated at 37 °C for 2 h in a shaking incubator, serially diluted, and plated on agar using the drop plate technique. After incubation at 37 °C for 12 h, colony-forming units (CFUs) were enumerated to assess bacterial survival. The tests were repeated 6 times for each group (*n* = 6).

### 2.8 *In vivo* Evaluation of the Wound Healing Capacity of the Bioactive PFMs in a Murine Model and Histological Staining

All animal procedures were approved by the Institutional Animal Care and Use Committee of the College of Staten Island. Dorsal hair of CD-1 mice (CD-1 IGS, Charles River Laboratories, USA) was removed using a depilatory cream (Nair, Church & Dwight Co., USA). Prior to application, all PFM samples were disinfected by UV irradiation. Following anesthesia and skin disinfection, a full-thickness excisional wound (∼1 cm diameter) was created on the dorsal surface using surgical scissors. Wounds were photographed daily for 18 days with a digital camera, and wound areas were quantified using ImageJ software (NIH, USA). Mice (n = 30) were randomly assigned to three groups: (i) control, treated with waterproof transparent dressings (CVS Health, USA); (ii) plain PLGA PFMs; and (iii) turmeric-loaded PFMs (0.5% turmeric/PLGA). Wound closure rate was calculated using the following equation.

$$\mathrm{wound closure rate}\;\left(\%\right)=\left[\left(A_{0}-A_{T}\right)/A_{0}\right]\times 100$$

where *A*_0_ = initial area of the wound at *t* = 0 and *A*_*T*_ = area of the wound on the day of observation.

A subset of mice was euthanized on day 13 postinjury, and the wounds along with adjacent skin were excised in full thickness. Tissue samples were fixed in 4% paraformaldehyde at 4 °C overnight, followed by cryoprotection in 20% sucrose at 4 °C for 24 h. The tissue samples were embedded in optimal cutting temperature (OCT) compound, frozen and cryosectioned at a thickness of 15 μm using a cryostat (CM1950, Leica Biosystems). Tissue sections were stained with hematoxylin and eosin (H&E) for general morphological evaluation and with Masson’s trichrome for collagen visualization. Stained sections were examined using a brightfield optical microscope (Axio Imager.A2, Carl Zeiss AG). Semiquantitative analysis of collagen deposition was performed by intensity measurement using the software ImageJ.

### 2.9 Pilot Study to Evaluate the Hemostatic Potential of the PFMs Using a Tail Vein Amputation Bleeding Model

The procedure was approved by the Institutional Animal Care and Use Committee of the College of Staten Island. To simulate venous rupture, a mouse tail amputation model was employed. CD-1 mice (CD-1 IGS, Charles River Laboratories, USA, 6 weeks old, 25–30 g) were randomly assigned to four groups (*n* = 5 per group). Anesthesia was induced via intraperitoneal injection of ketamine/xylazine (90/10 mg/kg). Using a sterile surgical scalpel, 50% of the tail length was amputated, and the tail was exposed to air for 10 s to allow normal initial bleeding. Blood was collected in a preweighed graduated cylinder to accurately quantify blood loss. The control group received no treatment following amputation. The bleeding tail was covered with cotton gauze in the second group. The third and fourth groups were covered with sterilized plain PLGA PFMs and 0.5% tur/PLGA PFMs, respectively. On average, the fabricated PFMs had a thickness of 0.30–0.35 mm. Blood was collected for 12 min. Bleeding was then monitored for 10 min postamputation to detect potential rebleeding events. The blood collected was weighed and recorded. The animals were then euthanized in accordance with the approved experiment protocols.

#### Statistical Analysis

Experimental data were analyzed using GraphPad Prism software. One-way and two-way analyses of variance (ANOVA) were applied as appropriate to assess differences among groups. A *P* value ≤ 0.01 was considered statistically significant. Data is presented as mean ± standard deviation (s.d.) for all the measurements. Tukey’s honestly significant difference (HSD) post hoc test was used to identify pairwise differences between group means.

## 3 Results and Discussions

### 3.1 Preparation of the PFMs by Solution Blow Spinning Process Using a Siphon-Fed Airbrush

Solution Blow Spinning (SBS) is a method of fiber fabrication that involves the utilization of two parallel concentric fluid streams. One stream comprises a polymer dissolved in a volatile solvent, while the other involves a pressurized gas flowing around the polymer solution.^36^ The integration of these two streams can be seamlessly achieved in a straightforward device or facilitated using a commercially available airbrush. Hence, this technique is also called “airbrushing”.^37^ In SBS, the solvent rapidly evaporates during fiber formation, allowing solid fibers to deposit directly onto the target surface. SBS has shown to be 10 times faster, and ∼100 times less costly to set up than electrospinning, and can deposit nanofibers on a wider variety of surfaces.^37^

While most studies have used gravity-fed airbrushes,^35,37^ this work employed a siphon-fed bottom-loading airbrush. This minimized the nozzle clogging during fabrication up to 2.5% w/v turmeric loading, thus allowing even the insoluble components of the turmeric to pass without disrupting the fiber formation process. This also enabled larger volumes to be utilized for fiber preparation at a time in comparison to the gravity-fed airbrushes.

The turmeric loadings (0.1%, 0.5%, and 2.5% w/v) were selected based on a preliminary screening of dispersion stability and solution blow spinning processability to define the feasible concentration window that provided consistent performance. Concentrations higher than 2.5% w/v resulted in rapid sedimentation and nozzle clogging, precluding continuous fiber fabrication. The 0.1–2.5% (w/v) turmeric loading window used in the study therefore reflects the optimized concentration range for consistently producing turmeric PFMs with minimal performance variation. The standard protocol involved adding turmeric powder to the PLGA/acetone solution, which was then magnetically stirred vigorously for 10 min and immediately processed. This ensured that acetone-soluble bioactive components of turmeric (e.g., curcuminoids, turmerone, ar-turmerone, and germacrone) were fully dissolved, while insoluble components were maintained as a uniform dispersion.

### 3.2 Mechanical Properties

The mechanical properties of PFMs containing varying concentrations of turmeric powder were assessed using dynamic mechanical analysis (DMA) with a film tension clamp. PFM thickness ranged from 0.30 to 0.35 mm across all samples, hence minimizing variations in cross-sectional area between samples. Tensile stress and Young’s modulus were calculated by normalizing the measured force to the macroscopic cross-sectional area of the specimens. Ultimate tensile strength was defined as the maximum value on the stress–strain curve, while Young’s modulus was calculated as the slope of the linear elastic region between 0 and 1% strain. Among the tested formulations, PFMs with 0.5% turmeric loading exhibited the highest ultimate tensile strength (0.80 ± 0.13 MPa), outperforming plain PLGA (0.33 ± 0.03 MPa), 0.1% turmeric loading (0.38 ± 0.03 MPa), and 2.5% turmeric loading (0.26 ± 0.05 MPa) (Figure 1). The Young’s modulus of the 0.5% turmeric-loaded PFMs was 0.33 ± 0.20 MPa. The mechanical properties of the optimum PFMs were close to fibrin, elastin (∼1 MPa) and some human tissues.^38,39^ The reported mechanical values represent apparent bulk properties of the porous fiber mats rather than intrinsic polymer properties.

**Figure 1:**
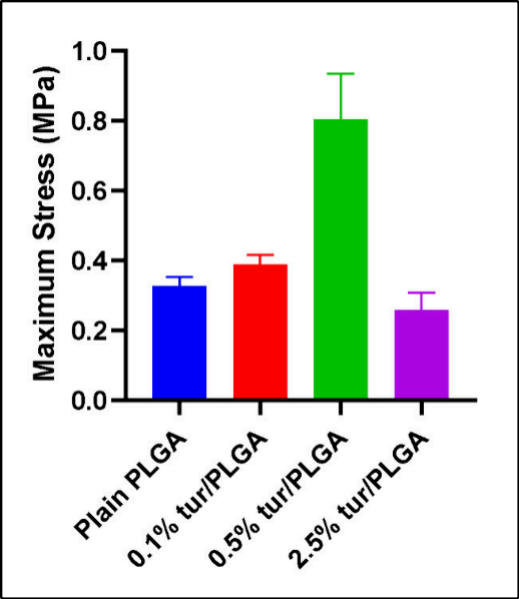
Comparison of maximum stress (MPa) of plain PLGA PFM and bioactive PFMs with varying turmeric concentrations. (*n* = 4, error bars indicate s.d.)

### 3.3 Surface Wettability

Surface wettability characteristics play an important role in protein absorption,^40^ cell adhesion^40,41^ and the adhesion of the PFMs to the wound bed.^42^ Better wetting of the mat surface will ensure stronger adhesion to the wound bed and better contact. Hydrophobic surfaces can hinder absorption of exudate and disrupt the balance of moisture required for optimal wound healing.^43^ Moderate surface wettability has been shown to be optimal for promoting wound healing.^44,45^

Contact angle analysis demonstrated notable differences in apparent wettability across the fiber formulations. Plain PLGA and 0.1% tur/PLGA PFMs showed similar contact angle values at both 0 and 60 s in PBS and hemoglobin (HGB) solutions, indicating minimal alteration in surface properties at low turmeric loading. In both cases, contact angles remained above 110°, confirming the predominantly hydrophobic nature of these surfaces (Figure 2A).

**Figure 2:**
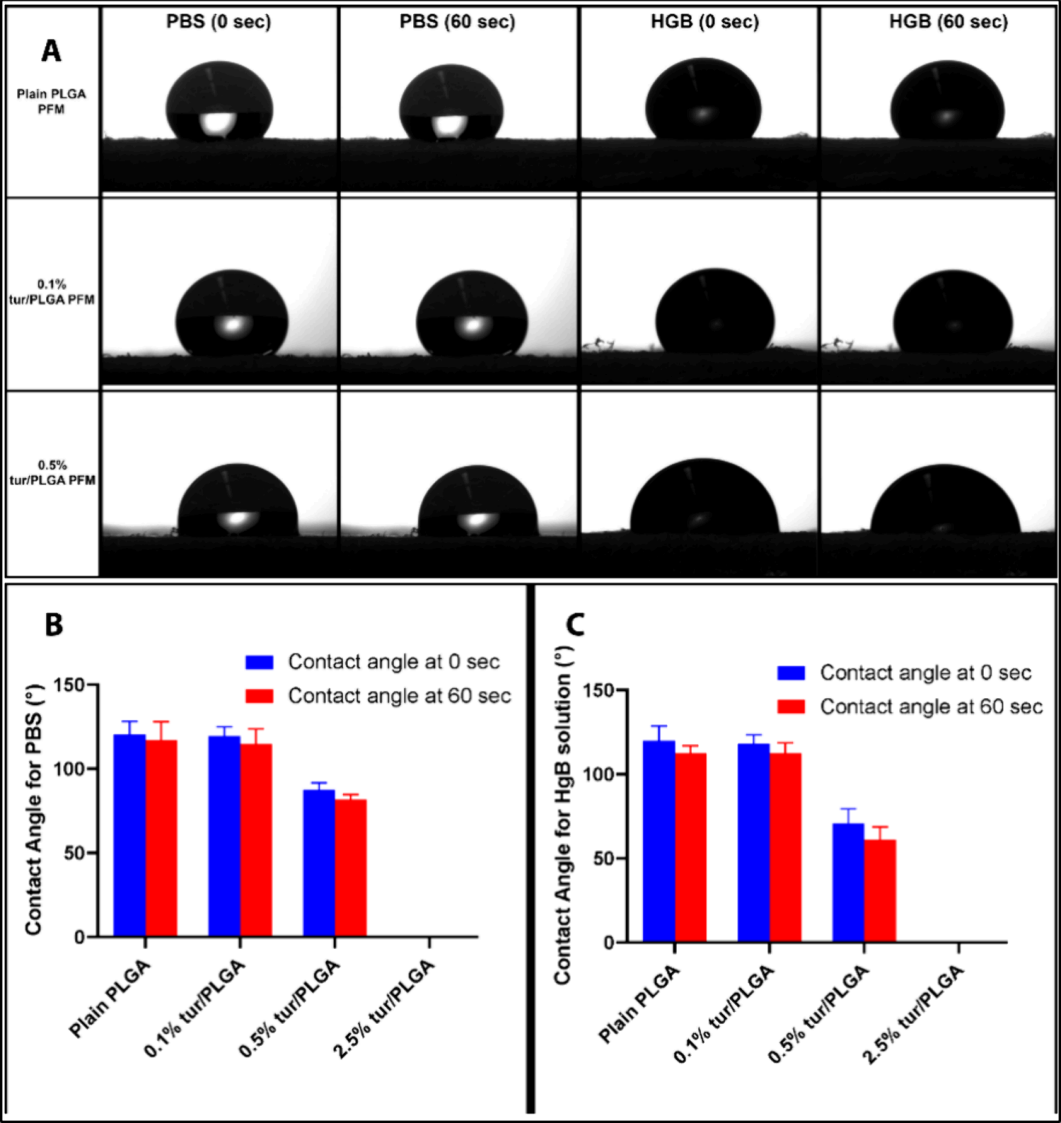
(A) Representative images of PBS and HGB solution droplets at 0 and 60 s on plain PLGA, 0.1% tur/PLGA and 0.5% tur/PLGA PFMs indicating the hydrophobic nature of plain PLGA and 0.1% tur/PLGA PFMs. (B) Contact angles of PFMs with different turmeric loadings at 0 and 60 s for PBS and (C) for HGB solution (*n* = 5, error bars indicate s.d.).

In contrast, the 0.5% tur/PLGA PFMs exhibited significantly improved wettability, with contact angles decreasing from 87.62 ± 4.01° to 81.58 ± 3.19° in PBS and from 70.78 ± 8.78° to 60.83 ± 7.83° in HGB solution over the 0–60 s interval (Figure 2A-C). This enhanced wettability can be attributed to the combined effects of surface chemistry and fibrous morphology. Partial surface enrichment of turmeric at this concentration likely alters the surface energy, while the inherent roughness and porosity of the fibrous mat amplify wetting behavior through Wenzel-type wetting and increased liquid spreading within the fiber network.^46^

At the higher turmeric loading (2.5%), stable contact angle measurements could not be reliably obtained, as both PBS and HGB solutions were rapidly absorbed into the mat. This immediate liquid infiltration could be attributed to the highly porous and interconnected fibrous morphology at high turmeric content, where capillary-driven wicking dominates over equilibrium surface wetting. In this case, the liquid uptake occurs faster than droplet stabilization, rendering apparent contact angle measurements very difficult. Loss of structural integrity of the mats due to liquid infiltration was also observed.

These findings identify the 0.5% tur/PLGA PFMs with moderate wettability as the optimal formulation for balancing fluid interaction and material integrity. The enhanced wettability observed at this concentration suggests superior fluid absorption and spreading, characteristics that facilitate nutrient transport and maintenance of a moist wound environment. Such properties are critical for accelerating wound closure and improving overall healing outcomes.^47^ Based on both mechanical properties and wettability, the 0.5% tur/PLGA formulation was selected as the optimal composition and was used for all subsequent material characterization, *in vitro* and *in vivo* experiments.

### 3.4 Water Uptake Capacity and Degradation Behavior

A moist wound environment is critical for promoting faster healing and improved tissue quality while reducing the risk of necrosis.^48^ Therefore, an ideal wound dressing should maintain a moist microenvironment at the wound site to optimize healing outcomes. However, most conventional dressings lack sufficient water-retentive capacity to achieve this.^49^ Water uptake increased progressively from 1 to 48 h in both plain PLGA PFMs and 0.5% tur/PLGA PFMs. Notably, 0.5% tur/PLGA PFMs exhibited significantly higher water retention, reaching a maximum of 126.45 ± 12.45% at 48 h, compared to 97.79 ± 6.20% for plain PLGA PFMs (Figure 3A).

**Figure 3:**
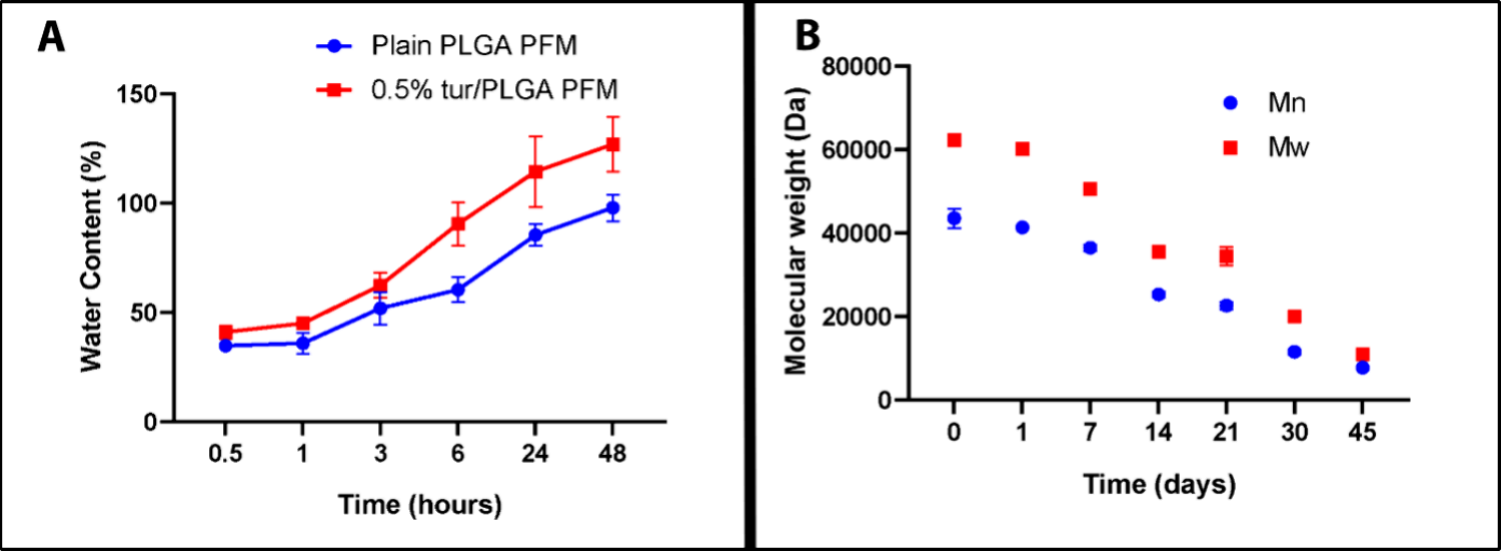
(A) Water uptake of the PFMs from 0.5 to 48 h (*n* = 6, error bars indicate s.d.). (B) Linear reduction of number-average and weight-average molecular weight of the 0.5% tur/PLGA PFMs in 45 days (*n* = 3, error bars indicate s.d.).

The number-average (Mn) and weight-average (Mw) molecular masses of the 0.5% tur/PLGA PFMs decreased linearly from 43.5 ± 2.4 kDa and 62.3 ± 1.3 kDa respectively at day 0 to 7.7 ± 0.3 kDa and 11.0 ± 0.6 kDa at day 45, respectively (Figure 3B). The polydispersity index (PDI) increased from 1.4 ± 0.1 to 1.8 ± 0.2 over the same period. No significant differences were observed between the 0.5% tur/PLGA and plain PLGA PFMs. Overall, Mn and Mw decreased by approximately 82%. The observed linear degradation profile is characteristic of surface erosion rather than bulk degradation.^50^ This observation is consistent with previous studies demonstrating that nanofibrous structures will exhibit degradation profiles distinct from those of bulk films.^51^ The high surface area-to-volume ratio of ultrafine fibers facilitates more efficient diffusion and neutralization of acidic degradation byproducts, thereby mitigating the autocatalytic effects commonly observed in bulk erosion.^35^

### 3.5 Surface Morphology and Fiber Diameter

Figure. [Fig fig4]A shows the photograph of 0.5% tur/PLGA PFM immediately after fabrication. The intense yellow color of the PFM is indicative of uniform incorporation of the acetone soluble curcuminoids. Under higher magnification, the SEM image of the PFM reveals the presence of the dispersed undissolved components of turmeric powder embedded in the matrix of the fibers (Figure 4B). Figure S1-A and S1–B from the Supporting Information further indicate a relatively uniform distribution of polydisperse turmeric particles (the acetone insoluble fraction) embedded within the fiber mat without any significant bulk particle agglomeration observed.

**Figure 4:**
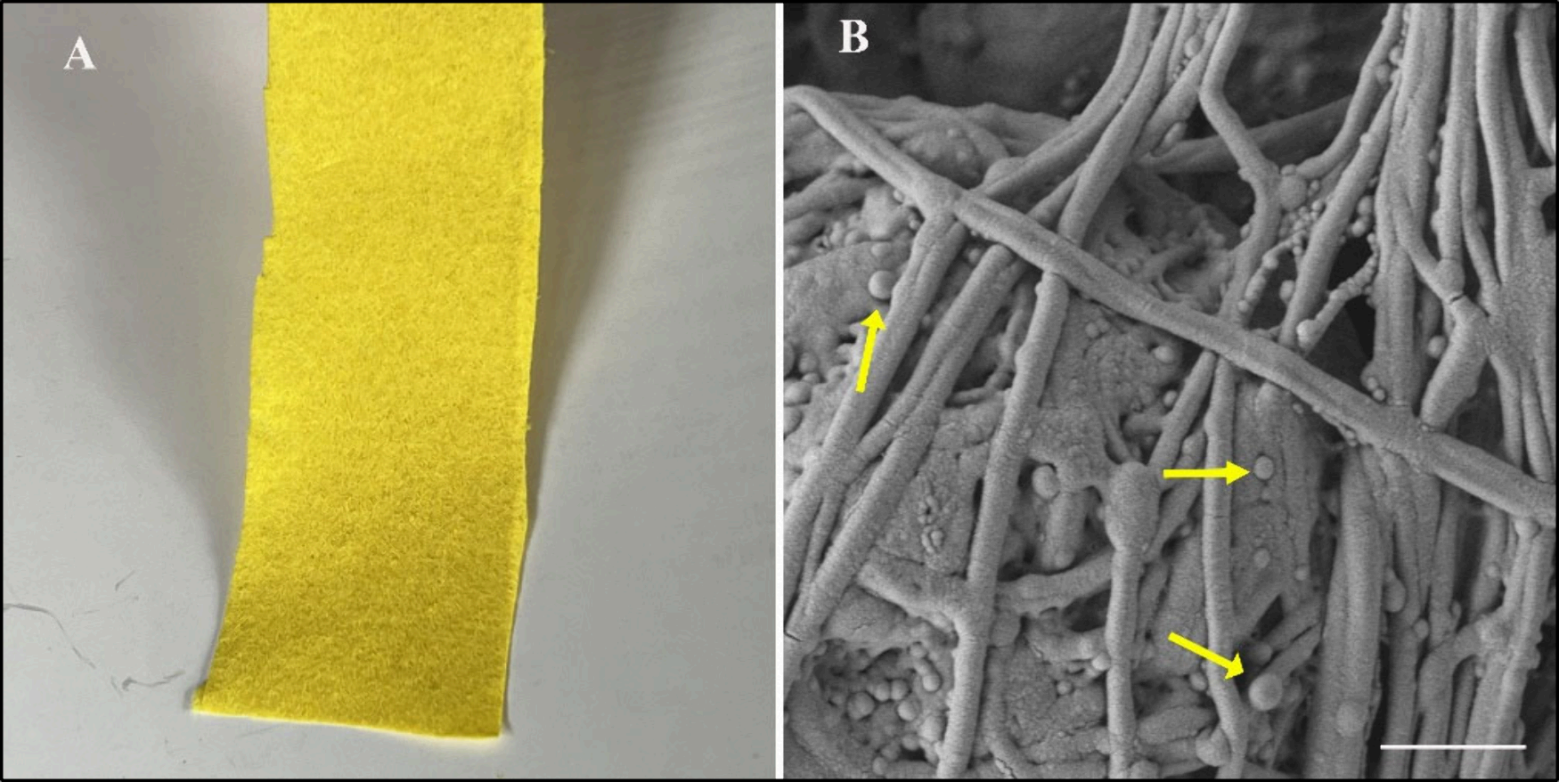
(A) A Photograph of 0.5% tur/PLGA PFM. The intense yellow color is consistent with the dissolution of the bioactive curcuminoids present in the turmeric and their uniform incorporation in the mat. (B) SEM image of the 0.5% tur/PLGA PFM. The presence of the dispersed undissolved components of turmeric powder embedded in fiber mat is clearly visible. Arrows indicate the undissolved components of the turmeric powder (Magnification = 5000×, Scale = 1 μm).

As shown in the image S1–C from the Supporting Information it can be observed that the incorporation of turmeric powder into the polymer matrix did not significantly alter the morphology and the fiber diameter of the PFMs. Plain PLGA fibers exhibited an average diameter of 296 ± 78 nm, while 0.5% turmeric-loaded PFMs measured 285 ± 67 nm. The fiber diameters were quantified using ImageJ software (NIH, USA) based on 60 randomly selected measurements per sample. These values fall within the diameter range reported for fibrin fibers, suggesting that the PFMs maintain the structural characteristics relevant to the native wound-healing environment.^52^

### 3.6 Antibacterial Effects of the PFMs

Turmeric-loaded PFMs significantly reduced the growth of *Escherichia coli* and *Staphylococcus aureus* after a 2 h exposure. Both species are frequently associated with surgical site infections, which can delay wound healing and contribute to the development of chronic wounds.^53,54^ Turmeric-loaded PFMs exhibited significant antimicrobial activity against both *E. coli* and *S. aureus* compared to plain PLGA PFMs and the untreated control (*P* ≤ 0.001). No significant differences were observed between plain PLGA PFMs and the untreated group, confirming that the antibacterial effect was attributable to turmeric. Relative to the untreated control, turmeric-loaded PFMs reduced colony-forming units by approximately 85% for *E. coli* and 96% for *S. aureus* (Figure 5). This finding mirrors the previous studies pertaining to the antibacterial properties of curcumin, the primary constituent of turmeric, as well as some of the other individual bioactive components present in the turmeric such as ar-turmerone, turmerone, curlone, germacrone, etc.^55–60^

**Figure 5:**
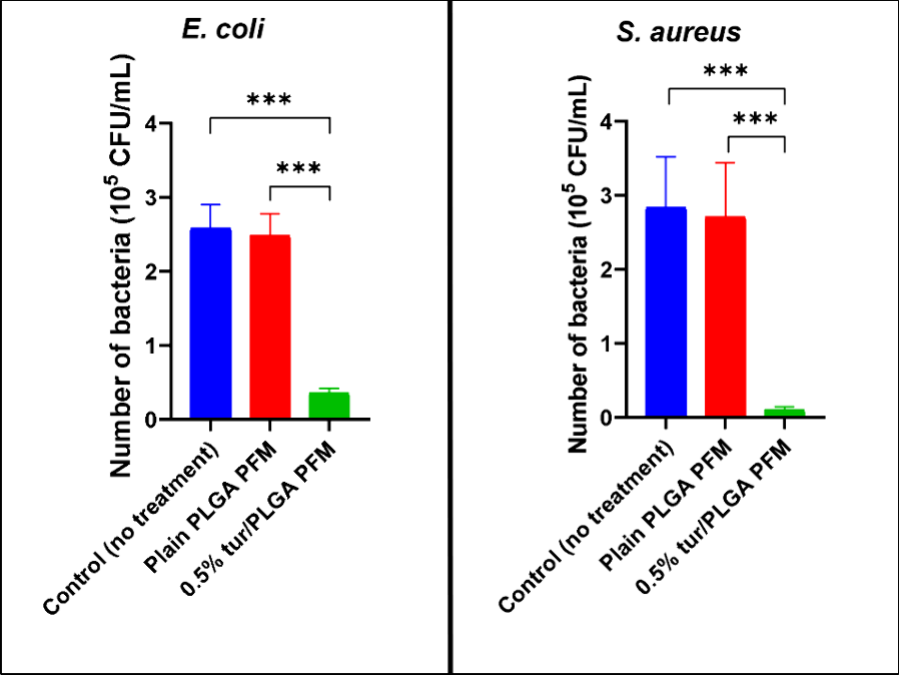
Reduction of number of bacteria after a 2 h treatment in 10^5^ CFU/ml (left, *E. coli*; right, *S. aureus* (*n* = 6, ***p ≤ 0.001, error bar indicates s.d.)).

### 3.7 Evaluation of Wound Healing on a Murine Model and Histological Analysis

Turmeric loaded PFMs accelerated wound closure and healing in a murine wound model. Turmeric loaded PFMs showed significantly faster wound closure in CD-1 mice when compared to plain PLGA PFMs and transparent dressings control (*P* ≤ 0.001). Figure 6A shows the macroscopic appearance of the wound at different time points postsurgery. The difference in healing rates was clearly observed after day 10. Turmeric PFMs showed the fastest healing rates with the wound closure of up to 93.8 ± 6.5% on day 15, therefore indicating almost complete wound closure. On the same day, the control group and plain PLGA PFMs showed the wound closure of 65.4 ± 5.7% and 73 ± 7.8% respectively (Figure 6B). In addition to accelerated wound closure, qualitative assessment revealed that the wounds treated with 0.5% tur/PLGA PFMs exhibited more mature granulation tissue formation and earlier re-epithelialization compared to the other groups. This is comparable to other systems which have utilized curcumin for treating wounds.^15,61^

**Figure 6:**
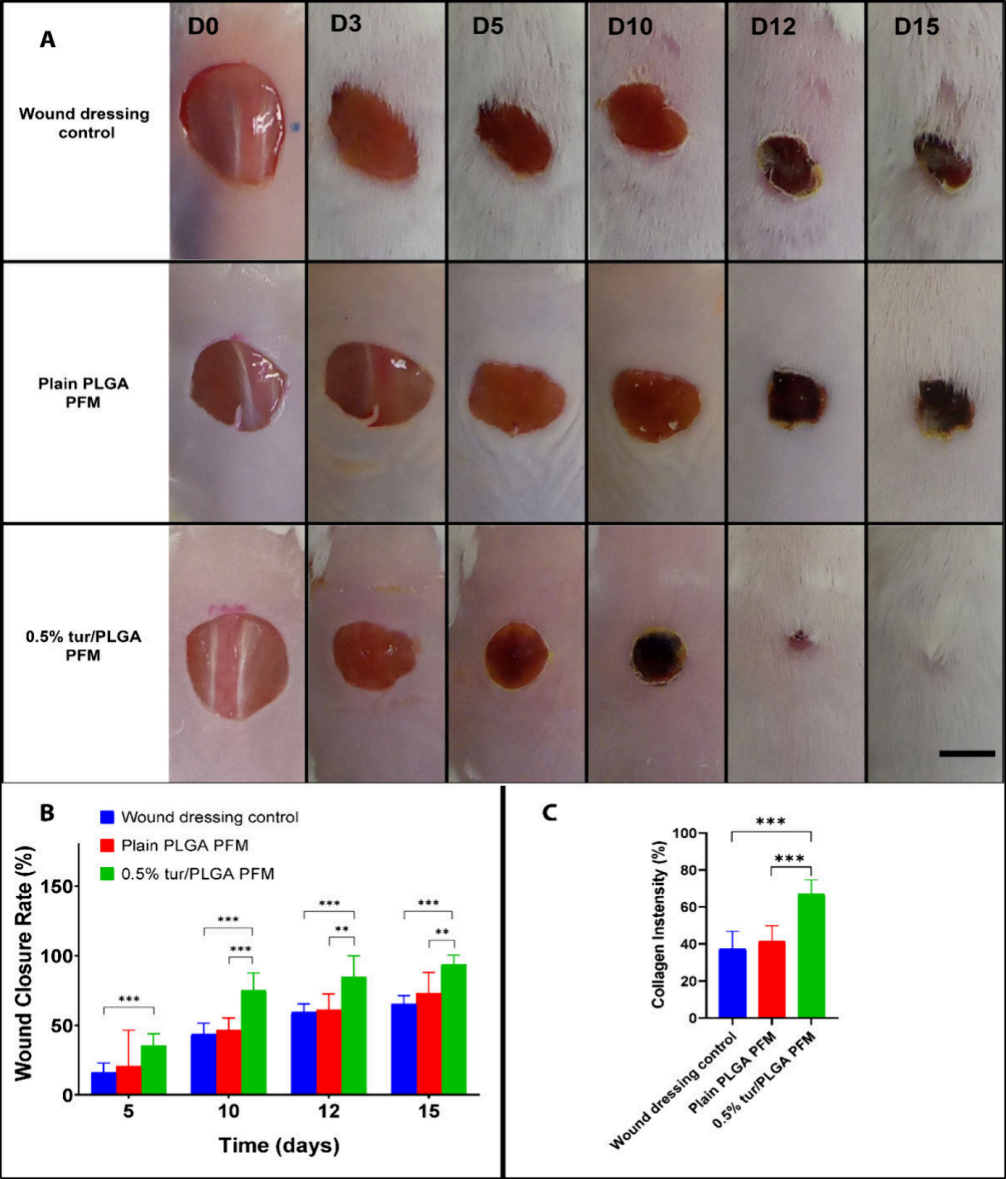
(A) Representative images of the wounds from day 0 to day 15 for the three groups (scale bar = 0.5 cm). (B) Wound area analysis for the three groups showing the progress in wound closure rate from day 5 to day 15 (*n* = 10, *** *p* ≤ 0.001, ** *p* ≤ 0.01, error bars indicate s.d.). (C) Difference in collagen intensity from 15 different fields of the same size (***p ≤ 0.001, error bars indicate s.d.).

Histological evaluation of wound sections from day 13 revealed clear differences in epidermal and dermal maturity, the granulation tissue quality and collagen deposition between the bioactive (0.5% turmeric) PFM group and the other groups (Figure 6C and Figure 7). The bioactive PFM group exhibited accelerated tissue maturation and the formation of a well-organized epidermis. The wound exhibited extensive re-epithelialization, characterized by a continuous, mature epithelial layer, minimal residual inflammatory infiltrate, and absence of significant eschar. Figure 7 C1 and C2. The control group and Plain PLGA group revealed a thick serum crust still attached to the wound bed with high concentration of inflammatory cells as seen in Figure 7 A1, A2, B1, and B2.

**Figure 7:**
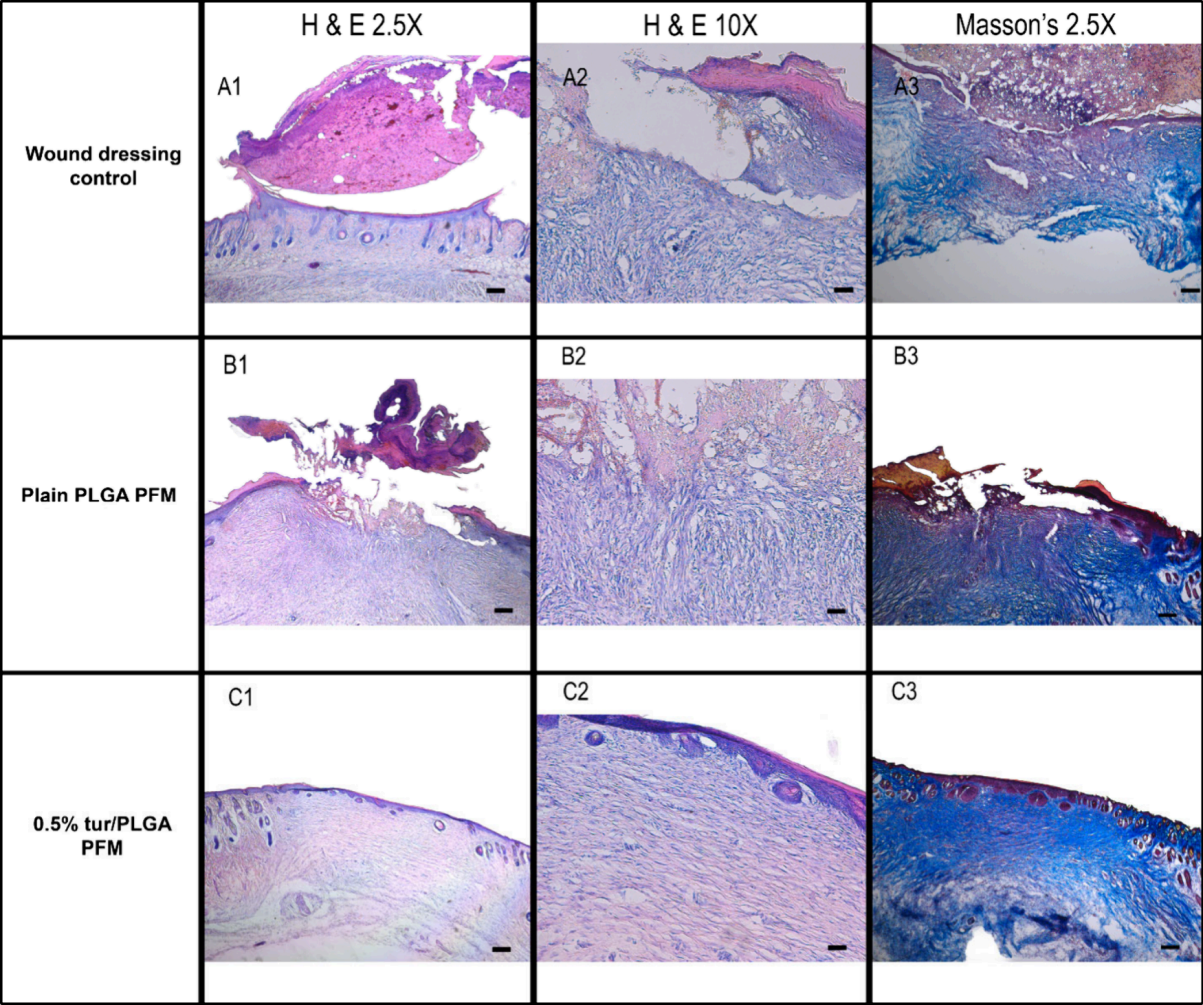
Bioactive PFMs enhances the formation of granulation tissue, collagen deposition and promotes accelerated wound healing. (A1, B1, and C1) Histological evaluation of the wound tissue from day 13 using Hematoxylin and Eosin (H&E) staining at 2.5× magnification. (A2, B2, and C2) H&E-stained wound tissue from day 13 at 10× magnification. (A3, B3, and C3) Masson’s Trichrome stained wound tissues from day 13 at 2.5× magnification (Scale bar = 200 μm).

Collagen deposition was assessed using Masson’s Trichrome staining of wound tissues collected on day 13. Wounds treated with the bioactive PFM exhibited well-organized, densely packed collagen bundles aligned parallel to the epidermis, indicative of advanced tissue remodeling and a mature stage of healing (Figure 7 C3). The dermal layer showed intense blue staining, reflecting the presence of highly structured, mature collagen closely resembling that of uninjured skin.

In contrast, collagen bundles in the wound dressing control group appeared haphazardly arranged and immature (Figure 7 A3). Wounds treated with plain PLGA PFMs exhibited some collagen deposition; however, the fibers were less homogeneous and poorly organized while displaying patchy, loosely arranged structures that lacked alignment with the epidermis (Figure 7 B3). Semiquantitative analysis of collagen deposition based on staining intensity at 15 representative fields of the same size revealed significantly higher collagen content in the bioactive PFM-treated group compared to both control groups (Figure 6C, *p* ≤ 0.001).

### 3.8 Tail Vein Amputation Study to Evaluate the Hemostatic Potential of the PFMs

The mechanism of action of many polymeric hemostats has been ascribed to physical and surface mediated mechanisms such as acting as sealant and a mechanical barrier rather than an active biological agent that activates the coagulation cascade.^35,62^ A pilot study using a tail vein amputation model was conducted to assess the hemostatic efficacy of the developed PFMs and to evaluate whether incorporation of turmeric powder adversely affects their hemostatic performance. The hemostatic behavior of the PFMs is primarily governed by physical interactions arising from their fibrous architecture and surface characteristics. Previous work has demonstrated that airbrushed PLGA nanofibers exhibit strong potential as surgical sealants, hemostatic agents, and buttress materials for tissue repair, and are capable of absorbing platelets and erythrocytes from citrated whole human blood.^35^ Aggregation of erythrocytes and platelets is an essential component of blood clot formation and bleeding control.^63,64^

Erythrocyte adhesion to fibrous surfaces is strongly influenced by surface wettability, with materials exhibiting water contact angles between 40° and 65° reported to promote cellular adhesion.^65,66^ In the present study, the PBS and HGB contact angles of the 0.5% turmeric/PLGA PFMs (bioactive PFMs) fell within this range, whereas plain PLGA PFMs exhibited contact angles exceeding 110° (Figure 2A–2C). This reduction in surface hydrophobicity is expected to enhance blood–material interactions and facilitate erythrocyte accumulation at the wound interface, contributing to effective mechanical hemostasis.

Consistent with this mechanism, the tail vein amputation assay showed that mean blood loss was highest in the open-tail group, followed by cotton gauze (20.4 ± 3.3 mg), plain PLGA PFMs (11.0 ± 4.2 mg), and was lowest for the bioactive PFMs (1.0 ± 0.6 mg) (Figure 8). One-way ANOVA revealed a significant difference among groups, and post hoc Tukey analysis demonstrated that both PFM groups differed significantly from the untreated and cotton gauze controls (*p* < 0.01). Although the difference between plain and bioactive PFMs was not statistically significant at this sample size, incorporation of turmeric at 0.5% (w/v) did not compromise the intrinsic hemostatic function of the fibrous mats and was associated with reduced mean blood loss, while potentially offering additional bioactivity relevant to wound healing and antibacterial activity. As an initial pilot investigation, this study was not intended to provide definitive mechanistic conclusions but rather to establish baseline hemostatic performance. The study provides initial evidence of hemostatic efficacy of these bioactive PFMs. Expanded studies to comprehensively assess the efficacy of the PFMs are in progress.

**Figure 8:**
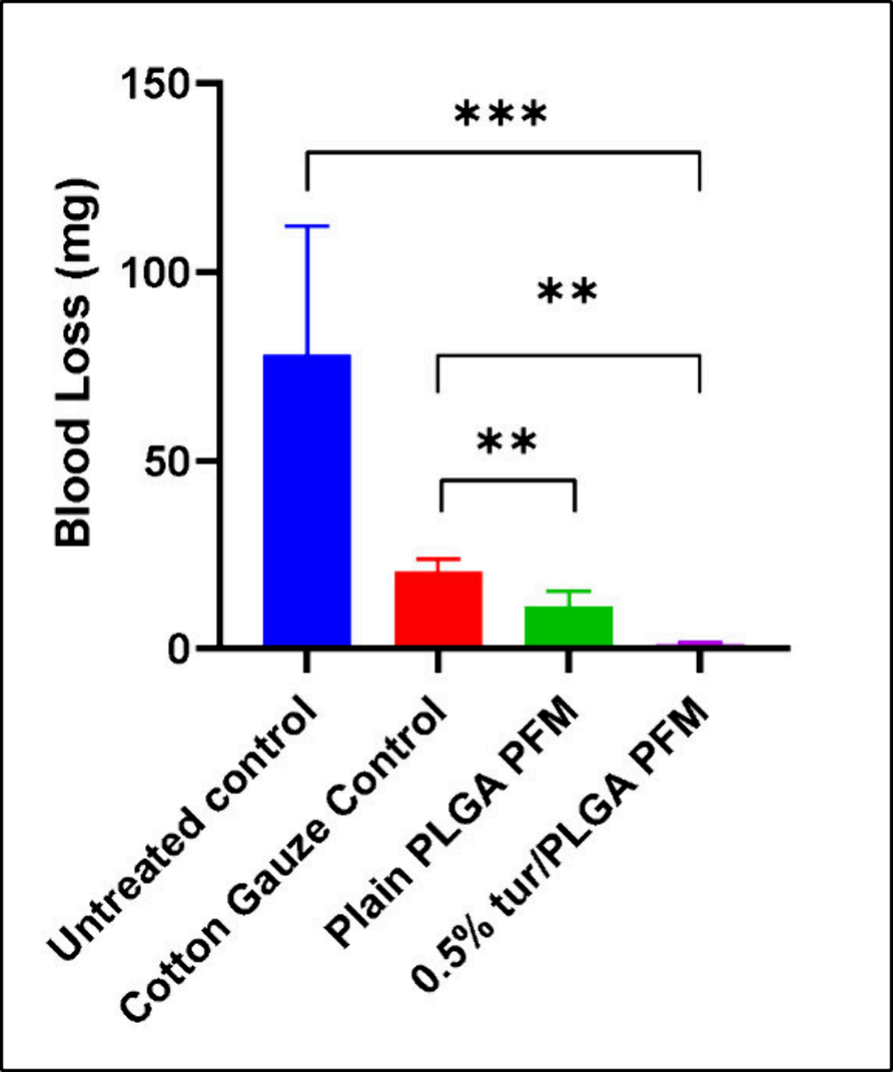
Blood loss in milligrams (*n* = 5, ***p ≤ 0.001, **p ≤ 0.01, error bars indicate s.d.).

## 4 Conclusions

The previously intractable formulation problem of producing a practical wound care product with the perennial wound care agent turmeric has been addressed in this study: A bottom loading airbrush loaded with turmeric in PLGA solution in acetone was used to produce bioresorbable turmeric/PLGA nanofibrous polymer fiber mats (PFMs). Among the formulations tested, the 0.5% tur/PLGA PFM was identified as the optimal composition based on a combination of mechanical and physicochemical properties. The optimized mat exhibited an ultimate tensile strength of 0.80 ± 0.13 MPa, comparable to fibrin-based soft tissue sealants (∼1 MPa). They also demonstrated enhanced wettability, with contact angles of 81.58 ± 3.19° in PBS and 60.83 ± 7.83° in hemoglobin solution therefore well suited for efficient fluid absorption and blood interaction in contrast to the more hydrophobic plain PLGA PFMs. SEM analysis revealed uniform ultrafine fibers with an average diameter of 285 ± 67 nm, and GPC measurements confirmed controlled degradation, with an ∼ 82% reduction in molecular weight over 45 days.

The 0.5% tur/PLGA PFMs demonstrated good antibacterial activity against *E. coli* and *S. aureus*, as well as significantly accelerated wound healing characterized by faster contraction, improved re-epithelialization, and mature collagen organization *in vivo*. A pilot tail-vein amputation study further indicated its potential to be used as a hemostatic material.

These findings highlight the feasibility of transforming turmeric into a bioactive, bioresorbable dressing that integrates structural support, antimicrobial efficacy, enhanced wound repair and hemostasis. This platform represents a promising candidate for a next generation wound care product with potential surgical and trauma applications. The present work focused primarily on a gauze-type format in which PFMs were deposited onto a glass substrate and subsequently removed to be utilized as free-standing mats. Ongoing studies are investigating the spray-on format, where turmeric-PFMs are generated directly on the wound surface to enable conformal, area-specific coverage. In addition, further comprehensive *in vivo* studies are planned to comprehensively characterize the hemostatic performance of these bioactive PFMs.

## Supplementary Material



## Notes

Additional scanning electron microscopy images of the 0.5% tur/PLGA PFMs at different magnifications showing the relatively uniform distribution of the polydisperse undissolved components of turmeric particles within the fibrous matrix and the high magnification image of the plain PLGA PFMs (PDF)
