# Supplementary material for: Turmeric Polymer Fiber Mats (T-PFMs): Solution Blow Spun Turmeric/PLGA Composites as a Comprehensive Wound Care Platform
Source: ACS Appl Polym Mater. 2026 Mar 24;8(7):4915–25. doi: 10.1021/acsapm.5c04766 (PMC13077634; doi:10.1021/acsapm.5c04766)
Supplement: Supplementary file 1 [file ap5c04766_si_001.pdf]

Supporting Information

Turmeric Polymer Fiber Mats (T-PFMs): Solution Blow Spun Turmeric/PLGA Composites as a Comprehensive Wound Care Platform

Kishan Kalluraya Yogesh[a,b], *Shannon Killen*[a], *Andrew Mancuso*[a,b], *Christina Viso*[a], *Joy Nasr*[a], *Kehinde Aiyegboyin*[a], *Victoria Cannava*[a], *Zaghloul Ahmed*[b,c,d] *& Krishnaswami S Raja*[a,b,e,f] *

[a] Department of Chemistry, The College of Staten Island, City University of New York, 2800 Victory Blvd, Staten Island, NY, United States 10314

[b] Graduate Center, City University of New York, 365 5th Ave, New York, NY, United States 10016

[c] Department of Physical Therapy, The College of Staten Island, City University of New York, 2800 Victory Blvd, Staten Island, NY, United States 10314

[d] Center for Developmental Neuroscience, The College of Staten Island, City University of New York, 2800 Victory Blvd, Staten Island, NY, United States 10314

[e] Institute for Macromolecular Assemblies, The College of Staten Island, City University of New York, 2800 Victory Blvd, Staten Island, NY, United States 10314

[f] Advanced Science Research Center Nanoscience Program, City University of New York, 85 St Nicholas Terrace, New York, NY, United States 10031

Corresponding author

*Krishnaswami S Raja, Department of Chemistry, The College of Staten Island, City University of New York, Staten Island, NY, United States. Email: Krishnaswami.Raja@csi.cuny.edu

Key Words: Turmeric, Polymer Fiber Mats, Wound Healing, Hemostatic, PLGA, Bioresorbable, Solution Blow Spinning

Scanning Electron Microscopy Images of the PFMs

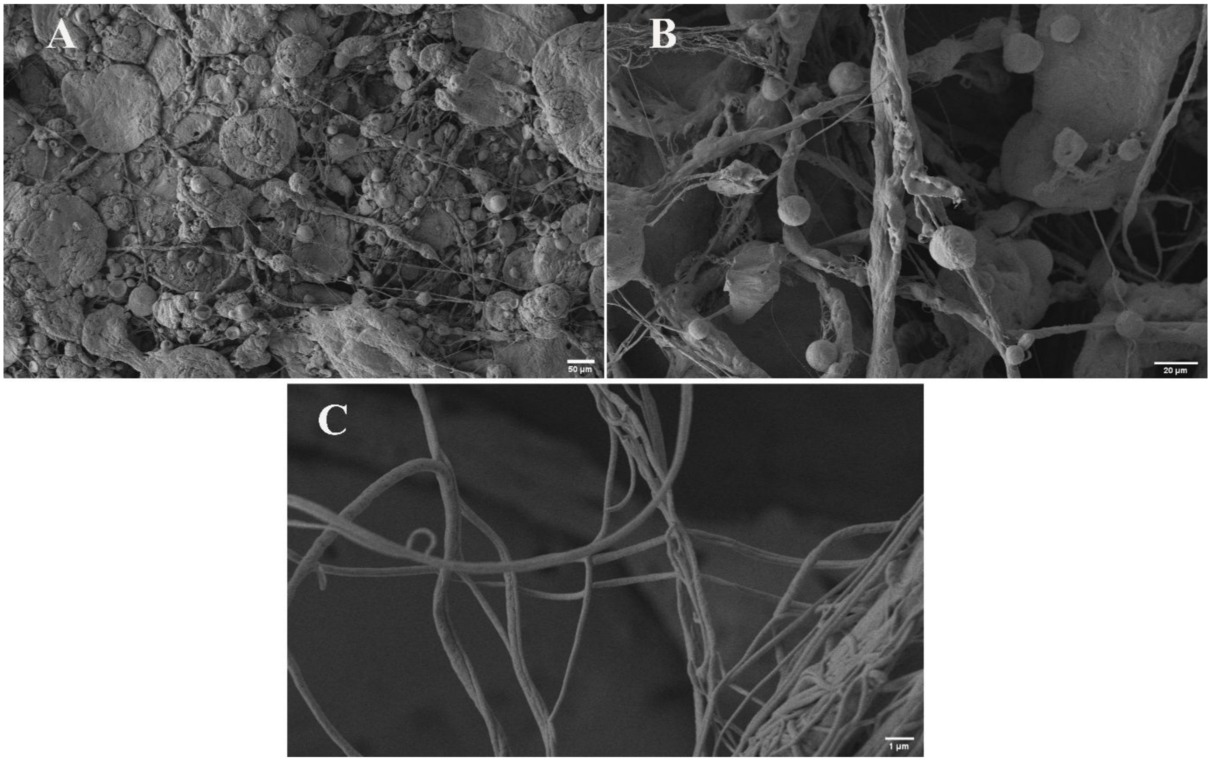

Figure S1. Scanning Electron Microscopy images. S1-A & S1-B) 0.5% tur/PLGA PFM at 100X and 400X magnification. S1-C) plain PLGA PFM at 5000X
